# A Novel Fragmentation Sensitivity Index Determines the Susceptibility of Red Blood Cells to Mechanical Trauma

**DOI:** 10.3389/fphys.2021.714157

**Published:** 2021-08-25

**Authors:** Elif Ugurel, Evrim Goksel, Polat Goktas, Neslihan Cilek, Dila Atar, Ozlem Yalcin

**Affiliations:** ^1^Research Center for Translational Medicine (KUTTAM), Koç University, Istanbul, Turkey; ^2^School of Medicine, Koç University, Istanbul, Turkey; ^3^Graduate School of Health Sciences, Koç University, Istanbul, Turkey; ^4^Centre for Applied Data Analytics Research (CeADAR), School of Computer Science, University, College Dublin, Dublin, Ireland

**Keywords:** supra-physiological shear stress, red blood cell fragmentation, oxidative stress, metabolic depletion, mechanical risk sensitivity, fragmentaton sensitivity index

## Abstract

Supraphysiological shear stresses (SSs) induce irreversible impairments of red blood cell (RBC) deformability, overstretching of RBC membrane, or fragmentation of RBCs that causes free hemoglobin to be released into plasma, which may lead to anemia. The magnitude and exposure tisme of the SSs are two critical parameters that determine the hemolytic threshold of a healthy RBC. However, impairments in the membrane stability of damaged cells reduce the hemolytic threshold and increase the susceptibility of the cell membrane to supraphysiological SSs, leading to cell fragmentation. The severity of the RBC fragmentation as a response to the mechanical damage and the critical SS levels causing fragmentation are not previously defined. In this study, we investigated the RBC mechanical damage in oxidative stress (OS) and metabolic depletion (MD) models by applying supraphysiological SSs up to 100 Pa by an ektacytometer (LORRCA MaxSis) and then assessed RBC deformability. Next, we examined hemolysis and measured RBC volume and count by Multisizer 3 Coulter Counter to evaluate RBC fragmentation. RBC deformability was significantly impaired in the range of 20–50 Pa in OS compared with healthy controls (*p* < 0.05). Hemolysis was detected at 90–100 Pa SS levels in MD and all applied SS levels in OS. Supraphysiological SSs increased RBC volume in both the damage models and the control group. The number of fragmented cells increased at 100 Pa SS in the control and MD and at all SS levels in OS, which was accompanied by hemolysis. Fragmentation sensitivity index increased at 50–100 Pa SS in the control, 100 Pa SS in MD, and at all SS levels in OS. Therefore, we propose RBC fragmentation as a novel sensitivity index for damaged RBCs experiencing a mechanical trauma before they undergo fragmentation. Our approach for the assessment of mechanical risk sensitivity by RBC fragmentation could facilitate the close monitoring of shear-mediated RBC response and provide an effective and accurate method for detecting RBC damage in mechanical circulatory assist devices used in routine clinical procedures.

## Introduction

Red blood cells (RBCs) are exposed to physiological shear stresses (SSs) of 5–20 Pa in the circulation, resulting in no RBC damage (Papaioannou and Stefanadis, 2005). Supraphysiological SSs in extracorporeal devices and artificial organs can overstretch the RBC membrane and lead to RBC fragmentation causing free hemoglobin (**Hb**) to be released into the plasma. RBCs are able to resist SSs up to 150 Pa for 100 s or 300 Pa for <1 s without being hemolyzed (Leverett et al., 1972; Baskurt and Meiselman, 2013b). RBC membrane may be ruptured and fragmented above this level of SS and exposure time, which is considered as a hemolysis threshold (Baskurt and Meiselman, 2012; Meram et al., 2013). However, the alterations in RBC membrane properties due to pathological conditions and environmental factors may significantly reduce this threshold (Baskurt and Meiselman, 2013a). The fragmentation of RBCs is documented in disorders such as hemolytic anemia and hereditary spherocytosis (Narla and Mohandas, 2017), independent of the turbulent shear flow generated in extracorporeal devices. The release of **Hb** from the overstretched or fragmented RBCs into the plasma may have a toxic effect on the cardiovascular system leading to hypertension, renal damage, and platelet activation (Minneci et al., 2005).

Schistocytes are defined as circulating RBC fragments (George and Nester, 2014). The presence of schistocytes in peripheral blood smears is critically important for the prognosis of thrombotic microangiopathy (Moake, 2002) and hemolytic uremic syndrome (Bagga et al., 2019). These RBC fragments are increased in cardiac prosthesis-related hemolytic anemia (Alkhouli et al., 2019) and mechanical heart valve recipients (Mecozzi et al., 2002), or during dialysis treatment (Pradhan et al., 2015; Schapkaitz and Mezgebe, 2017) and extracorporeal membrane oxygenation support due to mechanical damage (Mlinarić, 2016). The turbulence of flow and high supraphysiological shear forces are thought to be the causes of RBC fragmentation and hemolysis (Ellis et al., 1998). Although little or no hemolysis is detected in new generation extracorporeal devices, prolonged exposure to supraphysiological SSs leads to the mechanical damage of the membrane skeleton, may cause a loss in RBC deformability, or may reduce the hemolytic threshold in damaged RBCs (Gurbuz et al., 2004; Simmonds et al., 2014). Despite the “bio-safe” design of the new generation extracorporeal devices, oxidative stress (OS) is highly prevalent in the diseases indicated for these assist devices (Mcdonald et al., 2014). Excessive OS together with the effects of supraphysiological SSs significantly impairs RBC deformability (Mcnamee et al., 2018a) that may cause circulatory insufficiencies and propagate the development of the disease (Baskurt and Meiselman, 2003).

Shear stress under the hemolytic threshold changes the structural and mechanical properties of RBCs, which is considered as a subhemolytic damage (Baskurt et al., 2004). Subhemolytic damage is dependent on the level and the duration of shear stress-exposure as Simmonds and Meiselman showed that a 32 Pa SS exposure caused mild impairments in RBC deformability, but a dramatic impairment was recorded by a 64 Pa SS with only 4 s of exposure (Simmonds and Meiselman, 2016). The subhemolytic damage in RBCs might also be manifested by shortened life span *in vivo* (Brinsfield et al., 1962), increased trapping in the spleen (Sandza et al., 1974), decreased ability to change shape (Kameneva et al., 1999; Rampling et al., 2004; Marascalco et al., 2006; Relevy et al., 2008), increased aggregation and aggregability (Mcnamee et al., 2018b), morphological alterations with increased membrane roughness (Deng et al., 2018), or deterioration in the mechanical properties of the cells (Mcnamee et al., 2020). Unfortunately, these mechanisms are not evaluated routinely in patients supported by extracorporeal devices because there is no clinically applicable biomarker or approved methodology to detect subhemolytic RBC damage. In addition, the association between subhemolytic damage and the magnitude of SSs and their contribution to RBC fragmentation has not been fully elucidated. Therefore, in this study, we investigated RBC fragmentation, hemolysis, and alterations in RBC deformability by subhemolytic SSs in two different damage models (metabolic depletion (MD) and OS) and determined a novel sensitivity index for RBC fragmentation. The assessment of mechanical risk sensitivity of RBCs exposed to subhemolytic SSs could provide an accurate method to detect damaged RBCs in extracorporeal assist devices.

## Materials and Methods

### Blood Sampling

All blood samples were collected from the antecubital vein of healthy male and female volunteers aged between 20 and 40 years (*n* = 5). All experimental procedures used in this study were reviewed and approved by the Clinical Research Ethics Committee of Koc University (IRB: 020/2012). The use of human blood was in accordance with The Code of Ethics of the World Medical Association (Declaration of Helsinki), and all the participants gave written informed consent to participate in this study. Vacuum tubes containing EDTA (1.8 mg/ml) were used to obtain the blood samples (15 IU/ml). The hematocrit (Hct) of the blood samples was measured with a blood counter (ABX Micros 60 Hematology Analyzer, Horiba AbX, Japan) and set to 40% with autologous plasma. All the experiments were completed within 4 h following the blood sampling except the MD group.

### RBC Damage Models

#### Oxidative Stress Model

Oxidative stress *in vitro* was generated using hydrogen peroxide (30%; Sigma-Aldrich, cat. no. H3410). RBC suspensions (5% Hct) were incubated with sodium azide (2mM) at 37°C for 5 min prior to the peroxide addition. Hydrogen peroxide was added to the RBC suspension at a final concentration of 1 mM, followed by 25 min of incubation at 37°C. Incubated suspensions were washed at least three times with isotonic phosphate-buffered saline (PBS) solutions at 3,000 rpm for 5 min.

#### Metabolic Depletion Model

Blood samples with adjusted Hct at 40% were incubated on a tube roller for 48 h at room temperature. Measurements were taken on the same day of blood withdrawal (the 1st day) and after 48 h of incubation.

### Application of SS

A laser diffraction ektacytometer system (Laser assisted optical rotational cell analyzer; Lorrca MaxSis; RR Mechatronics, The Netherlands) was used to apply continuous SS of 5–100 Pa for RBC suspensions. The mechanism of the system has been previously described (Hardeman et al., 2001). Briefly, it contains a coaxial, coquette-type cylindrical shearing system. The inner cylinder has a laser that projects through the gap between the cylinders, with the resulting diffraction image digitally stored and analyzed. The RBC samples were mixed with polyvinylpyrrolidone (PVP) medium (viscosity: 29.6 mPa.s, Mechatronics, Hoorn, Netherlands) at a ratio of 1:200. RBC–PVP suspensions were subjected to SS by changing the rotation speed of the outer cylinder.

The following experimental procedure was followed for SS applications:

- The RBC suspensions were used to measure RBC deformability before continuous SS exposure at predetermined SS levels (i.e., 0.30–50 Pa)
- After the measurement was completed, the sample was aspirated from the gap and the cup was cleaned before the next sample was replaced.
- The gap was then filled with a new RBC suspension, and then, it was exposed to a constant 300-s long SS. A new RBC suspension was prepared for the application at each different level of SS (5, 30, 40, 50, 60, 70, and 100 Pa).
- Deformability of sheared RBCs was measured immediately after the SS application is ended.

### The Measurement of RBC Deformability

A laser beam was directed through the sample, and the resulting diffraction pattern was analyzed by the computer software. The diffraction pattern provides information of the elongation index (EI), which is calculated by the formula of (*A*–*B*)/(*A*+*B*), where *A* is the long axis and B is the short axis of the RBC. All measurements were obtained at 37°C. Maximum cell deformation at infinite stress (EI_max_) and the SS required to achieve one-half of this maximal deformation (SS_1/2_) were calculated from the deformability curves by the linear Lineweaver–Burke (LB) model (Baskurt et al., 2009). The curves were only fit to EI-SS data over the range of 1.65–50 Pa due to atypical behavior of RBCs at very low SS levels. RBC deformability was evaluated before and after the application of continuous SS. The susceptibility of RBC to mechanical trauma was analyzed according to the mechanical damage sensitivity formula (Simmonds et al., 2014). Briefly, it was evaluated by expressing the change in SS_1/2_:EI_max_ under each discrete preconditioning SS.

### Preparation of RBC Suspensions

The blood sample in the PVP medium was collected from the measurement gap of LORRCA and centrifuged at 300 × g for 5 min. Supernatant was collected for the measurement of plasma-free **Hb** concentration. RBC pellet was suspended in sodium phosphate buffer (PBS, Gibco, USA) at 40% Hct ratio. RBCs were suspended in PBS suspension with 1:40 dilution. Ten microliters of this suspension was further diluted in 20 ml filtered–sterile PBS and immediately measured by Multisizer 3 Coulter Counter.

### Determination of Plasma-Free Hb Concentration

The free **Hb** in the plasma mixed with PVP (29.6 mPa.s, Mechatronics, Hoorn, Netherlands) suspending medium was determined before and after SS applications by a modified cyanmet **Hb** technique (Lahet et al., 2008). Plasma with PVP was collected by centrifugation at 300 x g for 5 min. Supernatants were mixed with Drabkin reagent (1.1.3 mM potassium dihydrogen phosphate, 0.6 mM potassium ferricyanide, 0.8 mM potassium cyanide in 500 ml 30% Triton-X solution, and 1,100 ml distilled water). A **Hb** standard (Sigma-Aldrich, USA) was prepared with a concentration of 1 mg/ml in distilled water. Samples were incubated for 15 min in dark at room temperature. Absorbance values were recorded at 540 nm by a spectrophotometer (Hybrid Reader, Synergy H1, BioTek, VT, USA). The hemolysis index was calculated as previously described (Naito et al., 1994).

### The Measurements of RBC Fragmentation

The measurements of the cell volume and the cell count were performed by Multisizer 3 Coulter Counter (Beckman Coulter, Brea, CA, USA). This system contains two electrodes, one inside the aperture tube and one outside the tube. The outside electrode creates a current path through the electrolyte when an electric field is applied. The impedance between the electrodes is measured. The aperture, therefore, creates a “sensing zone,” and the particles suspended in the electrolyte can be counted by passing them through the aperture. When a particle passes through the sensing zone, a short-term change in the impedance is measured as a current pulse. The pulse height is proportional to the volume of the sensed particle. When a suspension is sucked into the aperture, each particle passing through the aperture can be recorded and digitized.

Each RBC suspension was studied in replicates. The distribution of the cell volumes was evaluated in the range of 0–30 fl (fragmented RBCs), 30–60 fl (RBCs in low volume range), 60–80 fl (RBCs in normal volume range), and 80–100 fl (RBCs in high volume range). Relative frequency was calculated by the percentage change of RBC count in the control group and damage models. Relative frequency vs. cell volume was calculated by a modified Gaussian approach (Bessman, 1988). The sensitivity of RBCs to fragmentation with the applied SS levels in different damage models is determined by the fragmentation sensitivity index (FSI) as described as follows:

$$\text{FSI}=\left({\text{CC}}_{\text{SS}}-{\text{CC}}_{\text{Before}}\right)/\;{\text{CC}}_{\text{Before}}^{*}100$$

where CC_SS_ is the cell count at defined SS level, and CC_Before_ is the cell count before preconditioning SS.

### Statistical Analysis

Results were recorded as mean ± SE, unless otherwise stated. All statistical analyses were performed with GraphPad Prism 7.0 (GraphPad Software Inc., La Jolla, CA, USA). The significance level was defined as *p* < 0.05. SS_1/2_ and EI_max_ values were calculated by LB approach (Baskurt et al., 2009). Plasma-free **Hb** concentration, deformability data, cell count data, and FSI were analyzed by one-way ANOVA followed by Tukey's multiple comparison test. Correlation between plasma **Hb** levels and fragmentation indices was analyzed by Spearman's test. Relative frequency data were analyzed by the two-way ANOVA, followed by Tukey's multiple comparison test. All experiments were performed in replicates and analyzed in a pairwise comparison.

## Results

### Plasma-Free Hb in Damage Models

**Figure 1:**
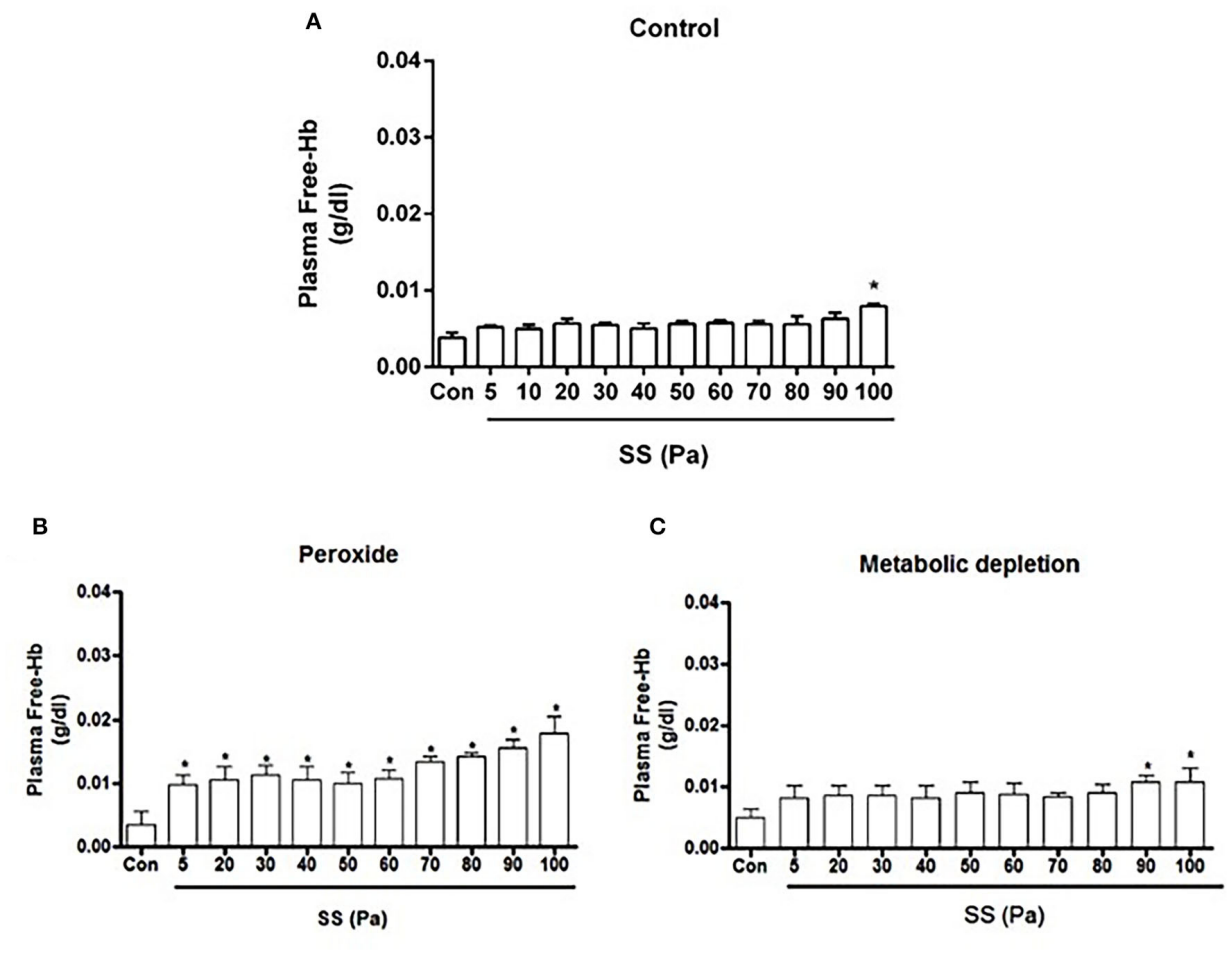
Free Hb concentration of healthy **(A)**, oxidative stress **(B)** and metabolic depletion **(C)** groups. Con: RBC sample before preconditioning SSs. Data are represented as mean ± SEM of *n* = 5. ^*^p < 0.05 refers to the difference from Con (one-way ANOVA).

Plasma-free Hb concentration significantly increased at 100 Pa level in untreated healthy RBCs (*p* < 0.05, Figure 1A). Free Hb was also detected in the MD model at 90 and 100 Pa levels of preconditioning SSs (*p* < 0.05, Figure 1C). In peroxide-treated RBCs (oxidative damage model), free Hb concentration significantly increased at each SS level (*p* < 0.05, Figure 1B).

### Red Blood Cell Mechanical Response to OS and MD

**Figure 2:**
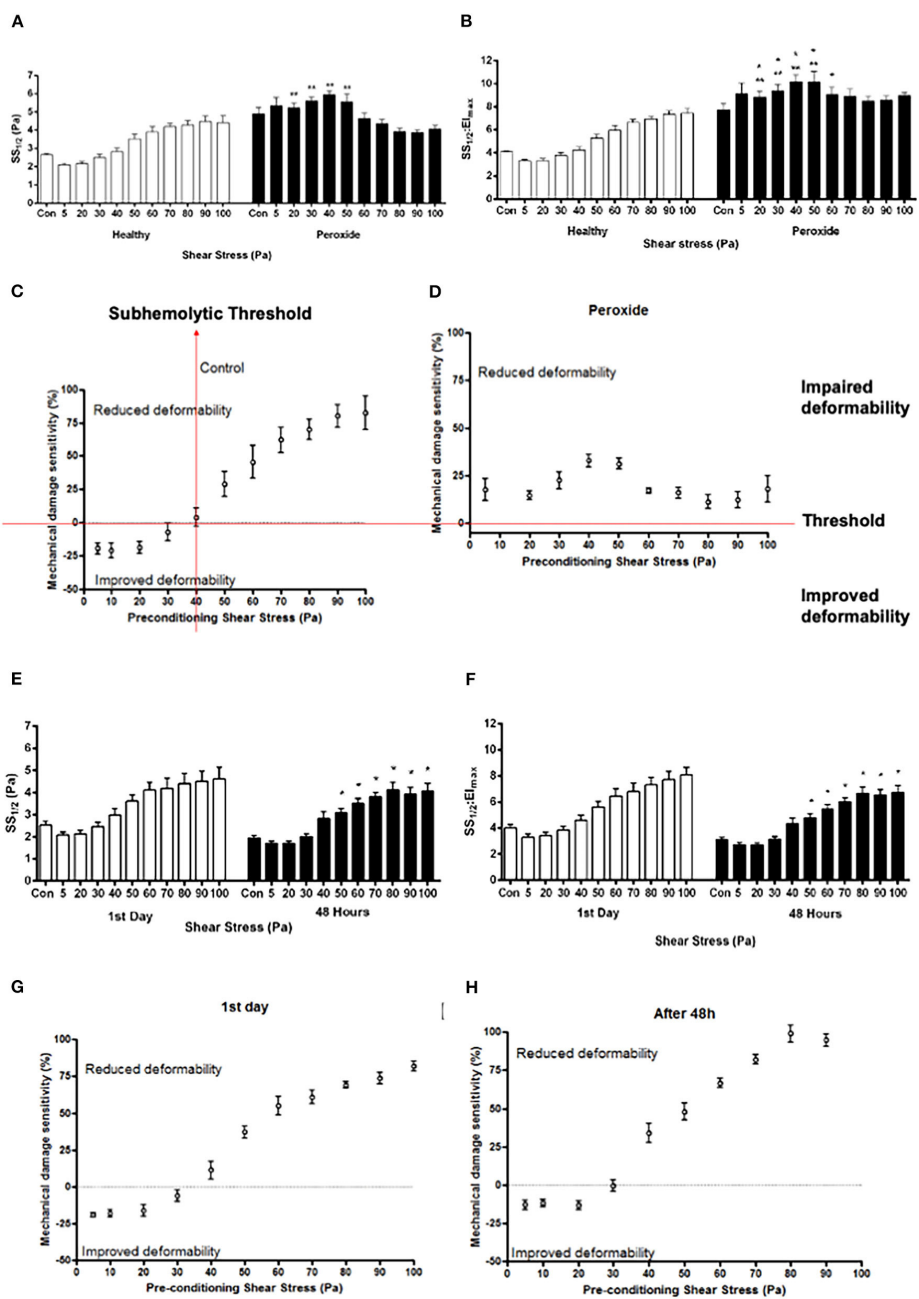
Curve-fit parameters that describe the elongation index (EI)–SS relationship for RBCs **(A, B, E, F)** and mechanical damage sensitivity to SS **(C, D, G, H)** exposed to 5–100 Pa for 300 s. Parameters shown in **(A,E)** are the SSs required for half-maximal deformation (SS_1/2_) and in **(B,F)** the ratio of SS_1/2_ divided by EI_max_. Panels **A, B, C**, and **D** represent oxidative stress (hydrogen peroxide), and panels **E, F, G**, and **H** represent metabolic depletion (48 h) groups. Con: RBC sample before preconditioning SS. Data are mean ±SEM. of *n* = 5. ^**^p < 0.05 refers to the difference from healthy group, and ^*^p < 0.05 refers to the difference from Con (one-way ANOVA).

The application of hydrogen peroxide significantly impaired the deformability compared with the healthy samples (*p* < 0.05, Figure 2). Hydrogen peroxide administration started to alter SS_1/2_ and SS_1/2_/El_max_ from 20 Pa SS level (Figures 2A,B, *p* < 0.05). However, after 50 Pa, the trend was reversed and SS_1/2_ and SS_1/2_/El_max_ values were decreased by the preconditioning SSs (*p* < 0.05, Figures 2A,B). The mechanical damage sensitivity index increased from 40 Pa level in healthy control samples. However, the susceptibility of hydrogen-peroxide-treated samples to mechanical damage increased at all applied SS levels (5–100 Pa), where the mechanical damage sensitivity index elevated compared with the healthy control samples (Figures 2C,D).

The SS_1/2_ and SS_1/2_:EI_max_ parameters in MD group were not significantly different than the first day group. However, the deformability of metabolically depleted samples was impaired in the range of 50–100 Pa compared with control samples before preconditioning SSs (*p* < 0.05, Figures 2E,F). RBCs on the first day were more tolerant to SSs than RBCs depleted for 48 h (Figures 2G,H). The sensitivity of metabolically depleted RBCs to mechanical damage increased from 30 Pa level (Figure 2H).

### Red Blood Cell Count and Volume Changes in Damage Models With Increasing SS

**Figure 3:**
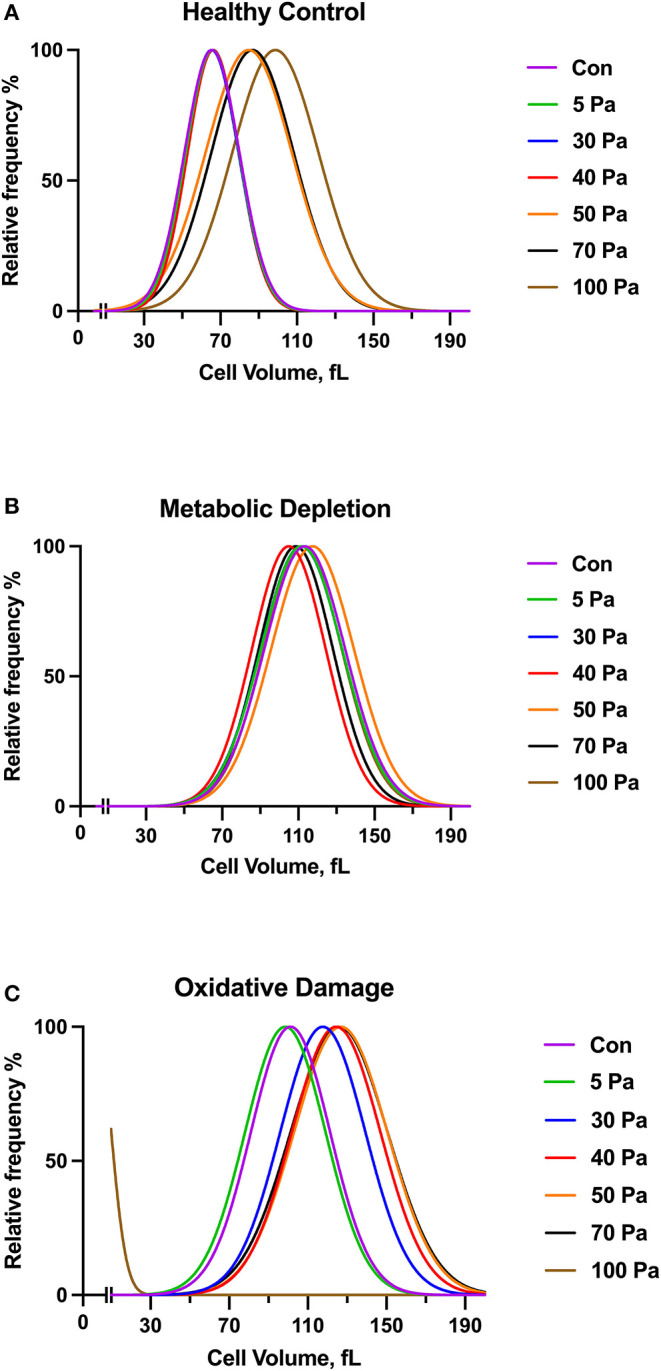
The curves show a volume distribution of healthy control **(A)**, metabolically depleted **(B)** and oxidatively damaged RBCs **(C)** with the range of SS (5–100 Pa), *n* = 5. No significant difference is recorded (Two-way ANOVA).

The volume of untreated healthy RBCs before preconditioning SSs was in the 30–110 fl range, where it peaked at 70 fl (Figure 3A). The curve of healthy RBCs preconditioned with 50 Pa SS was shifted to the right side of the graph, which shows an increase in RBC volume with applied SS level. The volume of RBCs further increased at 100 Pa level where it peaked at 110 fl. The relative frequency of metabolically damaged RBCs in different volume ranges was not affected by the SS level. Moreover, the damaged cells were already macrocytic compared with the healthy RBCs, where all curves of every SS level peaked at around 110 fl (Figure 3). The curve of oxidatively damaged cells before preconditioning SSs peaked at 100 fl, which indicates a volume increase by oxidative damage. Moreover, the volume of oxidatively damaged RBCs was altered by 30 Pa level as the curve was shifted to the right side and peaked at 120 fl. A second shift of the curve in oxidatively damaged cells was observed at 40 Pa SS, which showed that the volume increased above this SS level (Figure 3C). Supplementary Figure 1 shows that the volume of metabolically and oxidatively damaged RBCs was higher than the volume of untreated healthy RBCs. The largest RBC volume was observed in the MD group before preconditioning SS and at the level of 5 Pa SS (Supplementary Figures 1A,B). However, the effect of oxidative damage was more dominant on RBC volume from the level of 30 Pa (Supplementary Figures 1C–G). Standard deviation (SD) and area under curve (AUC) were also evaluated and provided in the Supplementary Table 1. Accordingly, SD and AUC of all groups increased with the increasing SS. This increment in SD and AUC indicates that the size of RBCs is more heterogeneous as RBCs are exposed to supraphysiological SSs. The curves of oxidatively and metabolically damaged cells had higher SD and AUC compared with healthy RBCs as damage models caused a heterogeneity among the RBC volumes. However, SD and AUC of oxidatively damaged RBCs drastically decreased at 100 Pa level (Supplementary Table 1) because oxidatively damaged cells were no longer tolerant to high SS at this level and became fragmented (Supplementary Figure 1G).

**Figure 4:**
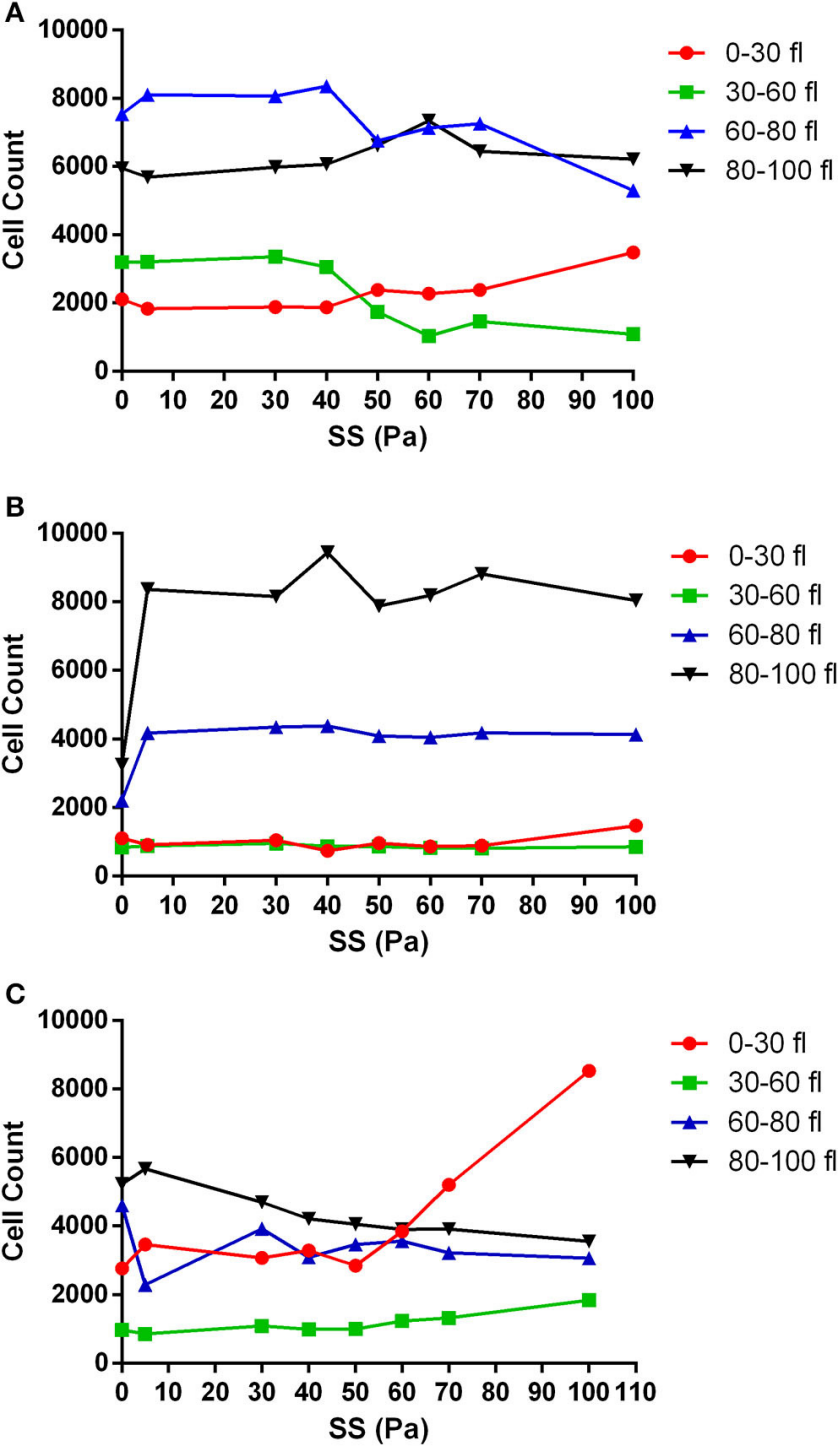
The change in the number of RBCs with applied SS in different volume ranges. **(A)** Healthy control RBCs, **(B)** metabolically depleted RBCs, and **(C)** oxidatively damaged RBCs. *n* = 5. 0–30 fl, 30–60 fl, 60–80 fl, and 80–100 fl ranges represent fragmented cells, smaller cells, normal cells, and larger cells, respectively. Significant differences are provided in Supplementary Table 2.

The volume of RBCs was divided into four different categories (0–30 fl, 30–60 fl, 60–80 fl, and 80–100 fl) to analyze the change in the cell count with different SS levels. RBCs in the 0–30 fl range represent fragmented cells. RBCs in the range of 30–60 fl were accepted as smaller cells. RBCs with normal cell volume fell in the 60–80 fl range. RBCs in the range of 80–100 fl range represent the cells with increased volume. The number of RBCs in the untreated healthy group started to change from the level of 40 Pa SS in all volume ranges (Figure 4A); however, this level was varied in metabolically depleted and oxidatively damaged RBCs (Figures 4B,C). The number of fragmented cells (0–30 fl) increased from 40 Pa level in healthy samples, which was accompanied by a decrease in the number of smaller and normal cells (Figure 4A). However, the number of fragmented cells slightly increased from 70 Pa level in metabolically depleted samples and drastically increased from 50 Pa level in oxidatively damaged samples. The pronounced elevation in the number of fragmented cells in OS group was accompanied by a decrease in the number of larger (80–100 fl) and normal (60–80 fl) cells. The number of fragmented cells was significantly different from the number of normal and larger cells in both healthy and MD groups (Supplementary Table 2). The difference between the number of fragmented and smaller cells in OS group was significant, which was not the case in healthy and MD groups (Supplementary Table 2).

### Red Blood Cell Fragmentation in Damage Models

**Figure 5:**
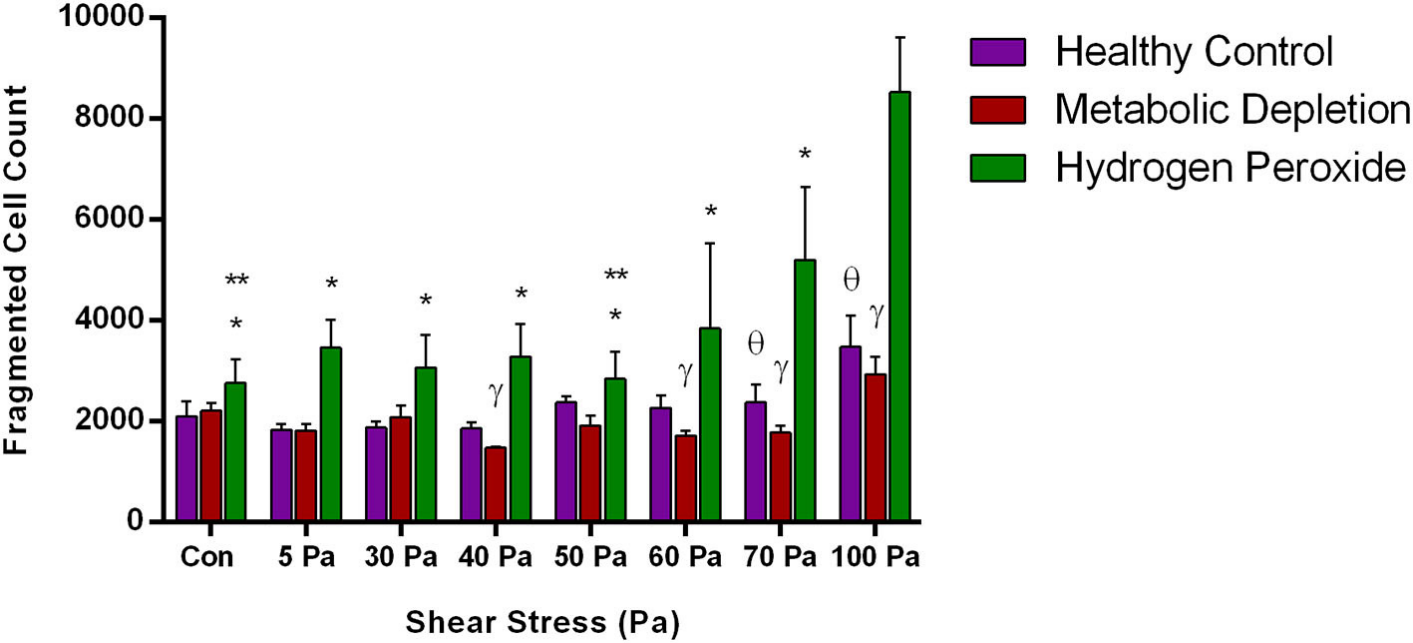
The change in the number of fragmented RBCs in the healthy control group and in the damage models with different levels of applied SS. Data are mean ± SEM of *n* = 5. ^*^p < 0.05, difference from 100 Pa; ^**^p < 0.05, difference from 70 Pa; γ *p* < 0.05, difference between metabolic depletion and oxidative damage groups; θ *p* < 0.05, difference between healthy control and oxidative damage groups (one-way ANOVA).

The number of fragmented RBCs was further evaluated in different damage models with increased SSs. Accordingly, fragmented RBC count was fluctuating in the healthy and MD groups in different SS levels; however, a relative increase at 100 Pa was observed (Figure 5). The number of fragmented cells was significantly higher in hydrogen peroxide-treated samples than in the other two groups at 70 and 100 Pa SS (*p* < 0.05, Figure 5). A significant increase in fragmented RBCs was observed at 70 and 100 Pa levels in the oxidative damage group compared with control samples before preconditionaning SSs (*p* < 0.05, Figure 5).

**Figure 6:**
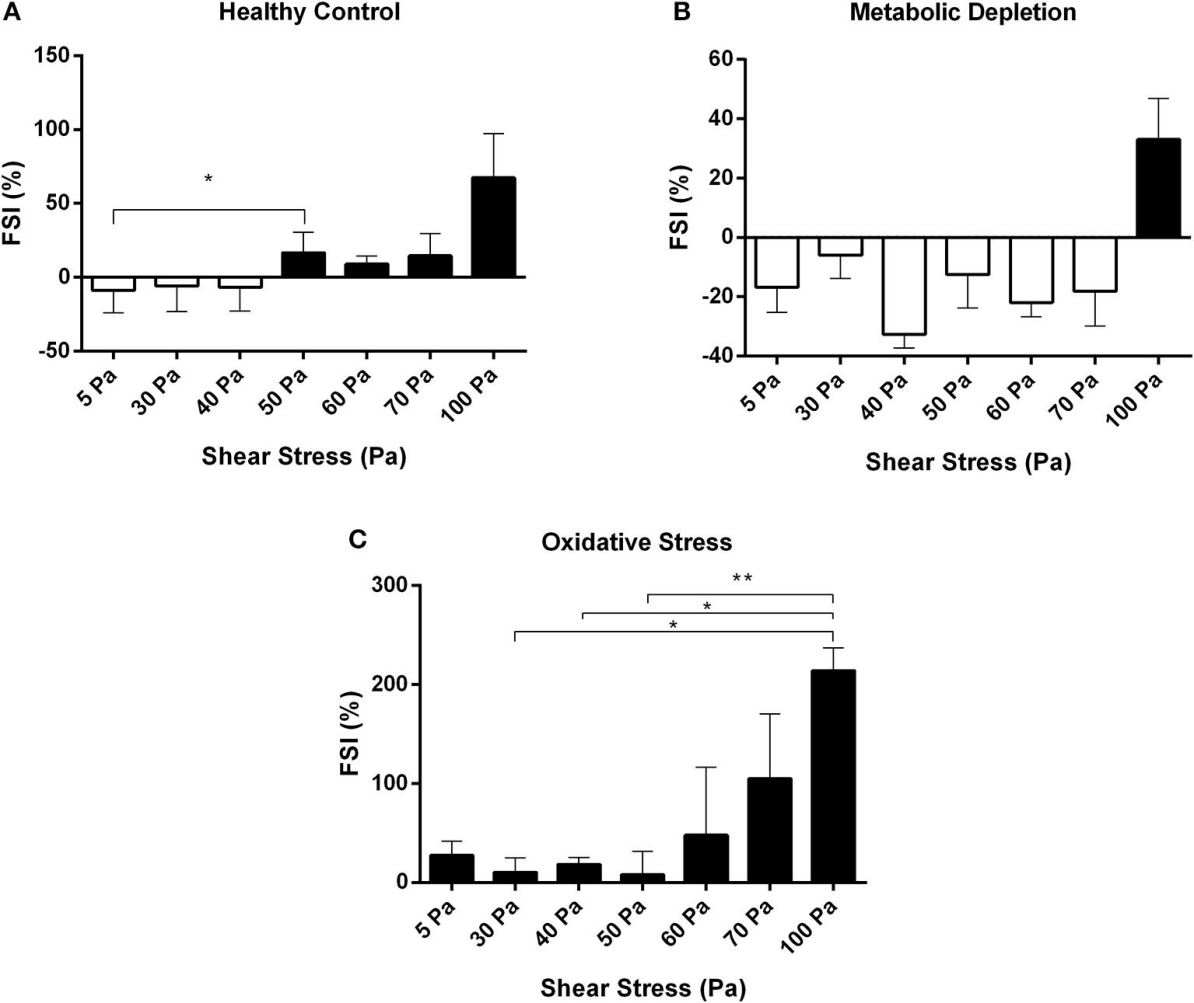
Fragmentation sensitivity index for healthy control **(A)**, metabolic depletion **(B)**, and oxidative damage **(C)** groups at different levels of SS. Black bars demonstrate the sensitive RBCs for fragmentation. Data are mean ± SEM of *n* = 5. ^*^p < 0.05, ^**^p < 0.0001 (one-way ANOVA).

The susceptibility of RBCs to fragmentation at different levels of SSs was evaluated by a novel FSI. Healthy RBCs were susceptible to fragmentation from the level of 50 Pa SS, which was significantly higher than the cells at 5 Pa SS (*p* < 0.05, Figure 6A). Healthy cells became more sensitive for fragmentation at 100 Pa SS (Figure 6A). FSI of metabolically depleted RBCs increased at 100 Pa (above zero level), which shows that depleted cells became sensitive to fragmentation from the level of 100 Pa SS (Figure 6B). However, oxidatively damaged RBCs were the most sensitive group for fragmentation that FSI increased above zero level at all applied SSs (Figure 6C). The most sensitive RBCs for fragmentation were observed at 100 Pa level in the OS group (*p* < 0.05, Figure 6C). We also correlated the fragmentation data with plasma-free Hb levels in healthy RBCs. Accordingly, Hb concentration is significantly correlated with the fragmented cell count (*r* = 0.762, *p* < 0.05) and with the FSI (*r* = 0.857, *p* < 0.05) (data not shown).

## Discussion

This study provides a novel sensitivity index for RBC fragmentation, which indicates the susceptibility of the cells to damage before they get fragmented. FSI shows that the healthy RBCs become more prone to fragmentation at 50 Pa SS level; however, this threshold decreases in the OS model. A prominent increase in the number of fragmented cells could be seen at 100 Pa level in healthy and metabolically depleted RBCs, whereas oxidatively damaged RBCs get fragmented at all SS levels (5–100 Pa) applied in this study. We also demonstrated that supraphysiological SSs increased the volume of RBCs, which is further increased by OS and MD.

Subhemolytic damage may occur by supraphysiological SSs below the hemolytic threshold. The detection of subhemolytic damage has been a focus of interest for decades due to a lack of a known biomarker or a measuring tool. Lee et al. showed that RBC deformability starts to deteriorate from the level of 30 Pa and suggested that deformability measurement can be a tool to show RBC subhemolytic damage (Lee et al., 2004). In this study, we found an impaired deformability in oxidatively damaged RBCs with an increasing SS_1/2_:EI_max_ in the range of 20 −50 Pa levels. An elevated SS_1/2_ and SS_1/2_:EI_max_ reflects an impaired deformability of RBC (Baskurt and Meiselman, 2013a). However, the decrease of SS_1/2_ after 50 Pa in oxidatively damaged RBCs would not indicate an improvement of deformability because the number of fragmented cells is increasing from this level of SS, which could intervene the deformability results. In addition, the mechanical sensitivity of oxidatively damaged RBCs increased from the basal level at all SS levels (Figure 2). The mechanical sensitivity increased up to 50 Pa SS and then no longer deteriorated. This could be because of the elevated fragmentation of oxidatively damaged RBCs from this level (Figure 4C). Similarly, SS_1/2_ and SS_1/2_:EI_max_ decreased from the level of 40 Pa in metabolically depleted RBCs, which may suggest an improved deformability. However, the volume of RBCs is drastically increased by MD and the effect of SS, which could limit maximal elongation. These results do not imply an improved deformability but rather an impairment in the mechanical properties of the cells.

The findings in this study demonstrated that RBC volume in OS and MD models prominently increased compared with the control group. According to a previous study, MD at room temperature significantly increased RBC volume in the first 24 h and further increased after 48 h, which is accompanied by a significant reduction in intracellular ATP levels (Reinhart et al., 2014). Energy depletion mostly affects ATP-dependent Na+-K+-ATPase pump on the cell membrane. The inhibition of this pump leads to cell swelling due to the accumulation of Na+ ions inside the cell (Giunta et al., 1992; Bak and Ingwall, 2003). Na+ ions contribute to extracellular osmolarity by 90%, and a significant decrease in extracellular osmolarity reflects an excess of water, leading to cell swelling (Lang et al., 1998). We have also observed elevated volume changes in oxidatively damaged RBCs, which might imply an increase in membrane Na+ permeability. Previous studies demonstrated that hydrogen peroxide administration resulted in an increased cell volume due to an increase in membrane Na+ permeability and Na+ influx, which would provide a gradient favoring water influx and an increase in cell volume (Schlenker et al., 2000; Simon et al., 2002). OS -induced Na^+^ influx may represent a beneficial adaptive response that restores cell volume over short periods, but sustained cation influx could contribute to the increase in intracellular Na^+^ and Ca^2+^, which is associated with progressive cell damage (Schlenker et al., 2015). On the other hand, we have observed an elevation in the volume of healthy RBCs (control group) at 50 and 70 Pa SS, followed by a further increase at 100 Pa SS level (Figure 3A). This would also suggest a Na^+^ influx by the effects of supraphysiological shear forces. According to a recent study, the volume response of RBCs to SS during a capillary transit, which is initiated by PIEZO1 channel activation, has a biphasic sequence of a rapid initial swelling followed by a slow cell shrinkage due to dehydration (Rogers and Lew, 2021). PIEZO1 channels are “sensors” of mechanical forces on RBC membrane and respond to SS by enabling Ca^2+^ influx and cell dehydration in coordination with Gardos channel (Coste et al., 2010; Cahalan et al., 2015). Rogers et al. suggested that PIEZO1 activation triggered CaCl_2_ influx and increased intracellular osmolarity, which was followed by a sharp decrease in extracellular osmolarity and generated a gradient for water influx into the cells (Rogers and Lew, 2021). Furthermore, a mechanosensitive TRPV2 channel was recently demonstrated by Belkacemi et al. in RBCs (Belkacemi et al., 2021). When the authors activated TRPV2 by channel agonists (cannabidiol and Δ9-tetrahydrocannabinol), they observed cell swelling and that the occurrence of stomatocytes and spherocytes increased and discocytes significantly decreased (Belkacemi et al., 2021). Although there are no data on the mechanical activation of TRPV2 in RBCs, Egee and Kaestner hypothesized that a strong activation of TRPV2 could lead to cell swelling by an increase in the intracellular Na^+^ concentration, which exceeds the K^+^ efflux (Egee and Kaestner, 2021).

The new generation automated hematology analyzers have included the parameter of fragmented red cells (FRCs) as a screening tool in the routine laboratory (Jiang et al., 2001; Lesesve et al., 2004). FRC parameter defines RBC fragments, which corresponds to the volume events smaller than 30 fl (Lesesve et al., 2004; Schapkaitz and Mezgebe, 2017). Although mean corpuscular volume of RBCs is between 80 and 100 fl (Maner and Moosavi, 2021), we analyzed the “normal” volume range of RBCs between 60 and 80 fl in order to extend the range toward a lower volume because others and we have demonstrated a reduction in RBC size measured by Multisizer instrument due to Coulter principle (Kim et al., 2019; Beckman Coulter, 2021). The unique discoid shape of RBCs denotes an inhomogeneous and a non-spherical geometry to the cell, which creates discrepancies in the electrical sensing zone (Eckhoff, 1967; Figueiredo, 2006). Therefore, we have evaluated the distribution of the RBCs in the range of 0–30 fl (fragmented RBCs), 30–60 fl (RBCs in low volume range), 60–80 fl (RBCs in normal volume range), and 80–100 fl (RBCs in high volume range). According to our findings, increasing levels of supraphysiological SSs slightly increased the number of fragmented cells in control and MD groups. The number of fragmented RBCs in the healthy control group increased after 40 Pa, which was accompanied by a decrease in the number of smaller and normal cells (Figure 4A). This finding is not surprising since RBC subhemolytic damage is revealed by the impairments in deformability at 30–40 Pa level (Simmonds et al., 2014). One might conclude that RBC deformability is deteriorated at supraphysiological SSs due to impaired membrane properties and increased cell volume, which are preceded by RBC fragmentation. The number of fragmented cells in control and MD groups is slightly increased at 100 Pa SS. However, fragmented cells are more abundant in the OS group, almost at all SS levels, and then drastically elevated at 70–100 Pa SS (Figure 5). Increased number of fragmented RBCs is also accompanied by plasma-free **Hb** levels found in this study. Accordingly, fragmentation occurs at 100 Pa in healthy RBCs, at 90–100 Pa in metabolically-depleted RBCs, and at 5–100 Pa in oxidatively damaged RBCs, which could explain the detectable hemolysis (Figure 1). The presence of fragmented cells is associated with plasma-free Hb levels and is indicative of hemolysis of mechanical origin (e.g., prosthetic heart valve, ECMO) or biological indicator of hemolytic disorders (Lesesve et al., 2001; Lou et al., 2014). Plasma **Hb** levels in this study are also positively correlated with the number of fragmented cells and the FSI.

The RBC fragmentation would be an indicator of irreversible cell damage due to mechanical trauma. We think that the detection and the measurement of the susceptibility of RBCs to fragmentation are of vital importance. Therefore, we propose a novel FSI in this study that enables the determination of the ratio of susceptible RBCs to mechanical trauma before they are fragmented. According to our findings, healthy RBCs are prone to fragmentation at 50 Pa SS without any detectable fragmented cells. The fragmentation sensitivity also increases by the ascending SS levels in healthy RBCs. MD increases RBC tolerance to mechanical stress where the FSI is elevated only at 100 Pa SS (Figure 6B). Prolonged incubation of RBCs in glucose-free buffer significantly reduces intracellular ATP levels so that dephosphorylation of membrane proteins is induced (Betz et al., 2009; Park et al., 2010). The interaction of the cytoskeleton with RBC membrane is enhanced by dephosphorylation, whereas it is decreased by the phosphorylation of spectrin or protein 4.1R (Manno et al., 1995, 2005). The strengthening of the interaction between RBC membrane and the cytoskeleton upon ATP depletion increases the surface tension of the membrane (Betz et al., 2009). According to a recent study, when such RBCs are exposed to a high pressure (200 MPa), fragmentation and hemolysis are greatly inhibited (Yamaguchi and Fukuzaki, 2019). This could explain the low fragmentation sensitivity of metabolically depleted RBCs in this study. On the other hand, the FSI increased at all SS levels (5–100 Pa) in the OS model (Figure 6C). The number of fragmented RBCs in oxidative damage model is increasing from the level of 50 Pa (Figure 4C), and almost all damaged RBCs are fragmented at 100 Pa SS level (Supplementary Figure 1G). Previous studies showed that OS increases RBC sensitivity to shear-mediated damage (Mcnamee et al., 2018a). In this study, we demonstrated that the RBC sensitivity for fragmentation due to mechanical stress is impaired even at physiological SS levels (e.g., 5 Pa) in oxidatively damaged RBCs. We expected that healthy RBCs would be more resistant to fragmentation; however, they are prone to fragmentation at 50 Pa SS, which is a considerably low level compared with the mechanical stresses that occur in circulatory assist devices (Selgrade and Truskey, 2012). The detection of the susceptibility of RBCs to fragment before they undergo fragmentation would be a valuable approach to evaluate the sensitive RBCs to mechanical trauma. The FSI proposed in this study will enable us to determine the fragility of RBCs exposed to mechanical stress prior to fragmentation and hemolysis. The assessment of RBC fragmentation would provide an effective and accurate method for detecting subhemolytic RBC damage in patients supported by mechanical circulatory assist devices and in pathological conditions where RBCs are more prone to fragmentation.

## Data Availability Statement

The datasets presented in this study can be found in online repositories. The names of the repository/repositories and accession number(s) can be found in the article/Supplementary Material.

## Ethics Statement

The studies involving human participants were reviewed and approved by the Ethics Committee of Koç University, School of Medicine (IRB:020/2012). The patients/participants provided their written informed consent to participate in this study.

## Author Contributions

EU, EG, PG, NC, and DA conducted the experiments and performed the data analysis and wrote the manuscript. OY supervised the experimental study design and interpretation. EU, EG, PG, NC, DA, and OY read and approved the final version of the manuscript. All authors contributed to the article and approved the submitted version.

## Author Disclaimer

The content is solely the responsibility of the authors and does not necessarily represent the official views of the Presidency of Strategy and Budget.

## Conflict of Interest

The authors declare that the research was conducted in the absence of any commercial or financial relationships that could be construed as a potential conflict of interest.

## Publisher's Note

All claims expressed in this article are solely those of the authors and do not necessarily represent those of their affiliated organizations, or those of the publisher, the editors and the reviewers. Any product that may be evaluated in this article, or claim that may be made by its manufacturer, is not guaranteed or endorsed by the publisher.

## Supplementary Material

Supplementary Figure 1 The histograms show a volume distribution of healthy control (purple curves), metabolically depleted (green curves), and oxidatively damaged (pink curves) RBCs before preconditioning with SS **(A)** and after preconditioning with 5 Pa **(B)**, 30 Pa **(C)**, 40 Pa **(D)**, 50 Pa **(E)**, 70 Pa **(F)**, and 100 Pa **(G)** SS.

Supplementary Table 1 Distribution width of RBC volume at different SS levels.

Supplementary Table 2 Significant differences between different volume ranges in healthy and damaged RBCs.
