# Supplementary figures and images for: A Novel Fragmentation Sensitivity Index Determines the Susceptibility of Red Blood Cells to Mechanical Trauma
Source: Front Physiol. 2021 Aug 25;12:714157. doi: 10.3389/fphys.2021.714157 (PMC8424113; doi:10.3389/fphys.2021.714157)

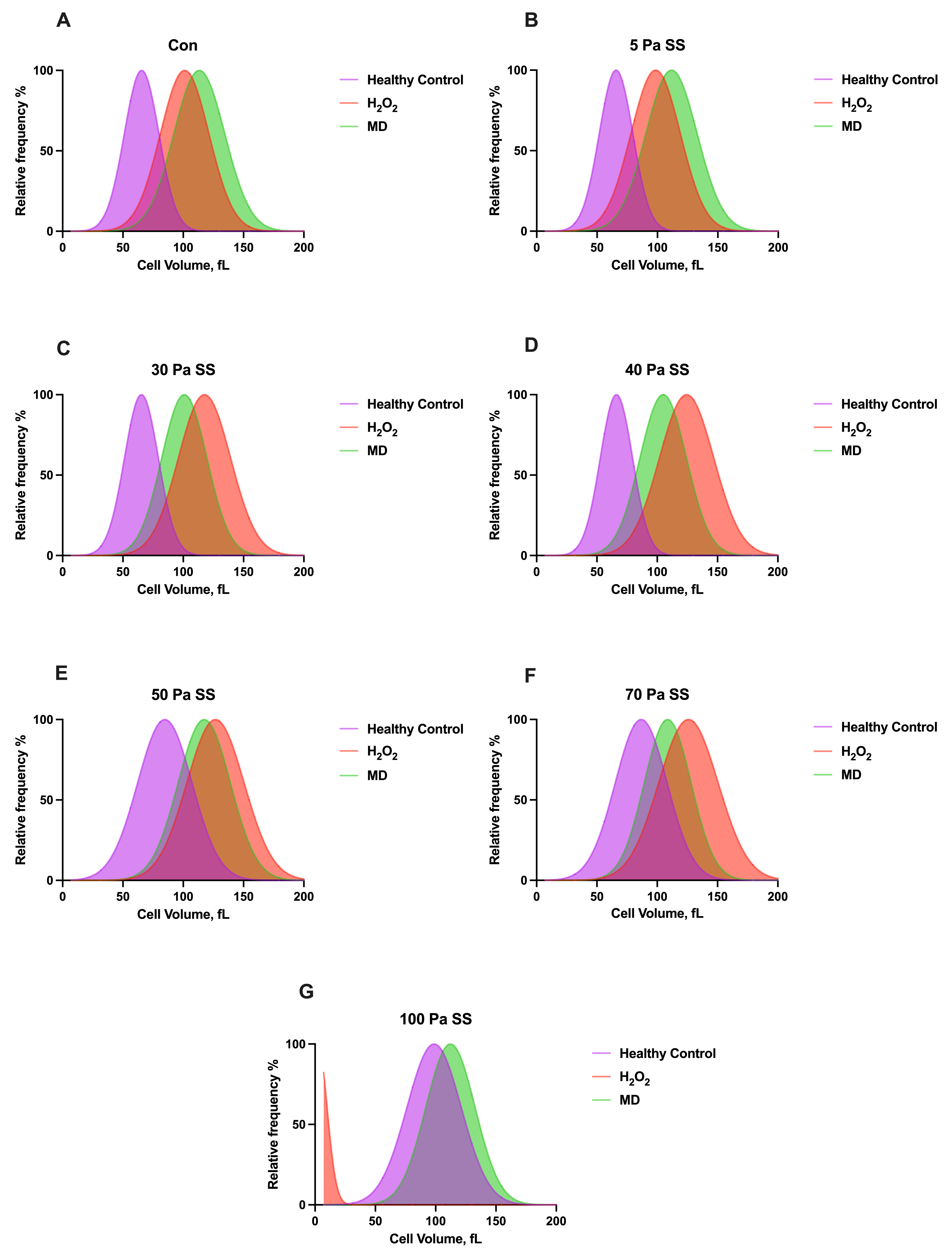

Supplement: Supplementary Figure 1 — The histograms show a volume distribution of healthy control (purple curves), metabolically depleted (green curves), and oxidatively damaged (pink curves) RBCs before preconditioning with SS (A) and after preconditioning with 5 Pa (B), 30 Pa (C), 40 Pa (D), 50 Pa (E), 70 Pa (F), and 100 Pa (G) SS. [file Image_1.tiff]
