# Supplementary material for: A Novel Fragmentation Sensitivity Index Determines the Susceptibility of Red Blood Cells to Mechanical Trauma
Source: Front Physiol. 2021 Aug 25;12:714157. doi: 10.3389/fphys.2021.714157 (PMC8424113; doi:10.3389/fphys.2021.714157)
Supplement: Supplementary Table 1 — Distribution width of RBC volume at different SS levels. [file Table_1.DOCX]

**Supplementary Table 1.** Distribution width of RBC volume at different SS levels

|  | **Control SD** | **Control AUC** | **Peroxide SD** | **Peroxide AUC** | **MD SD** | **MD AUC** |
| --- | --- | --- | --- | --- | --- | --- |
| **Control** | 14,4 | 3609,927 | 20,19 | 5061,418 | 21,19 | 5312,108 |
| **5 Pa** | 13,96 | 3499,623 | 20,52 | 5144,146 | 21,27 | 5332,163 |
| **30 Pa** | 14,11 | 3537,227 | 21,7 | 5439,959 | 21,31 | 5342,191 |
| **40 Pa** | 13,42 | 3364,251 | 22,58 | 5660,566 | 19,34 | 4848,332 |
| **50 Pa** | 22,6 | 5665,580 | 23,8 | 5966,407 | 21,7 | 5439,959 |
| **70 Pa** | 21,56 | 5404,863 | 24,68 | 6187,014 | 19,43 | 4870,894 |
| **100 Pa** | 22,57 | 5658,059 | 7,637 | 1914,514 | 20,47 | 5131,611 |

SD: Standard deviation, AUC: Area under curve, MD: Metabolic depletion
