# Supplementary material for: A Novel Fragmentation Sensitivity Index Determines the Susceptibility of Red Blood Cells to Mechanical Trauma
Source: Front Physiol. 2021 Aug 25;12:714157. doi: 10.3389/fphys.2021.714157 (PMC8424113; doi:10.3389/fphys.2021.714157)
Supplement: Supplementary Table 2 — Significant differences between different volume ranges in healthy and damaged RBCs. [file Table_2.DOCX]

**Supplementary Table 2.** Significant differences between different volume ranges in healthy and damaged RBCs.

|  | **Healthy RBCs** | **Metabolically depleted RBCs** | **Oxidatively damaged RBCs** |
| --- | --- | --- | --- |
| 0-30 fl vs. 30-60 fl | ns | ns | p<0.01 |
| 0-30 fl vs. 60-80 fl | p<0.001 | p<0.0001 | ns |
| 0-30 fl vs. 80-100 fl | p<0.0001 | p<0.001 | ns |
| 30-60 fl vs. 60-80 fl | p<0.0001 | p<0.0001 | p<0.001 |
| 30-60 fl vs. 80-100 fl | p<0.001 | p<0.0001 | p<0.001 |
| 60-80 fl vs 80-100 fl | ns | p<0.001 | p<0.001 |

ns: Not significant
